# Modelling cognitive flexibility with deep neural networks

**DOI:** 10.1016/j.cobeha.2024.101361

**Published:** 2024-06

**Authors:** Kai Sandbrink, Christopher Summerfield

**Affiliations:** Department of Experimental Psychology, University of Oxford, Oxford, UK

## Abstract

Neural networks trained with deep reinforcement learning can perform many complex tasks at similar levels to humans. However, unlike people, neural networks converge to a fixed solution during optimisation, limiting their ability to adapt to new challenges. In this opinion, we highlight three key new methods that allow neural networks to be posed as models of human cognitive flexibility. In the first, neural networks are trained in ways that allow them to learn complementary ‘habit’ and ‘goal’-based policies. In another, flexibility is ‘meta-learned’ during pre-training from large and diverse data, allowing the network to adapt ‘in context’ to novel inputs. Finally, we discuss work in which deep networks are meta-trained to adapt their behaviour to the level of control they have over the environment. We conclude by discussing new insights about cognitive flexibility obtained from the training of large generative models with reinforcement learning from human feedback.

## Introduction

Natural environments place heterogeneous demands on an organism. Flat terrain gives way to rocky hillsides, plain sailing to choppy waters or chit-chat to strenuous political debate. Biological lifetimes are short, and organisms cannot learn to expect every challenge that a volatile world may throw up. In psychology and neuroscience, it has long been proposed that brains have evolved tailored mechanisms for *cognitive flexibility*, that is, computational processes designed to deal with a changeable world through on-the-fly task prioritisation [1–3]. Psychological theories of cognitive flexibility often invoke a dual-process framework in which resource-in-tensive control systems (housed in prefrontal cortex) can be mobilised to suppress habitual behaviours when environmental demands grow. For example, patients with prefrontal damage are mostly untroubled by routine tasks but falter when required to innovate [4]. In neural recording studies, task contexts that are prone to incur errors or conflict activate putative monitoring mechanisms in medial prefrontal or cingulate cortices [5,6]. Envisaging tabular implementations of reinforcement learning (RL) models, neuroscientists have equated cognitive flexibility with ‘model-based’ processes that exploit a state transition matrix to mentally simulate possible future outcomes (rather than relying on cached value estimates from experience history) [7,8]. Model-based inference may also require prefrontal circuits [9].

Cognitive flexibility is required in tasks where different contexts require different rules. In primates, rapid switching between tasks is facilitated by the formation of explicit neural codes for rules, and computational models that are equipped by hand with rule-coding neurons can account for empirical patterns of task switching and error-related adjustment [10]. If they are invariant to context, rule neurons can also allow agents to generalise over physically dissimilar stimuli with overlapping selection demands. Computational models that draw inspiration from neural architectures and algorithms have shown how abstract rule neurons can emerge during training, and support generalisation at test. Rule neurons emerge most readily when systems are endowed with computational modules that resemble the Prefrontal Cortex (PFC), and use motifs (such as selective gating) that are hallmarks of primate executive function [11].

However, the recent renaissance in connectionist accounts of perception and cognition [12,13] invites us to consider how notions of cognitive flexibility can be incorporated into computational models based on deep learning systems. At first, the two notions might seem incompatible, because neural networks are trained to *converge* — to find a fixed point in parameter space where the task is satisfied, and beyond which no policy change is warranted. So, can networks learn to be cognitively flexible by gradient descent? If so, what constraints on optimisation may be required? Or are notions of mental flexibility largely chimeric, emerging naturally as undifferentiated neural networks are incrementally trained? In this review, we present three approaches that describe how simple changes to inductive biases at the level of training distribution and network architecture can induce neural networks to exhibit behaviour that is surprisingly flexible.

## Dual-process deep reinforcement learning

Deep learning systems rely on gradual tuning of network parameters to satisfy a cost function. Where the objective is to maximise scalar reward, a deep neural network is trained to either approximate the optimal value function [14], or to directly learn an optimal policy [15]. In the former approach, states are typically buffered in a form of ‘episodic’ memory and replayed in tandem with ongoing experience to stabilise training [16], in a process often thought of as echoing the dialogue between hippocampus and neocortex [17]. Here, flexibility arises from the way fast and slow memory processes jointly contribute to value learning [18]. By contrast, the latter alternative, known as the policy gradient approach, uses a deep network to learn a policy *π* (a distribution of actions given states) that maximises reward, often complementing this ‘actor’ with a separate ‘critic’ network that estimates state values. In neuroscience, policy gradient networks have been used to model perceptual decisions [19] and task switching [20] but have gained limited traction as general-purpose theories of cognition, perhaps because they are computationally intensive and technical to implement.

RL has roots in control engineering, the field that derives optimal policies for controlling a dynamical system (e.g. a plant) to meet a specified goal (e.g. maximise output and minimise cost). A canonical idea in control theory is that an optimal policy can be approximated via two quantities, one that specifies the cost *q* (*x*) for a given state *x*, and the other a divergence between the control dynamics *p* (*x*′|*x, u*) and passive dynamics *p* (*x*′|*x*), which are the goal-conditioned and default transition matrices, respectively, for a given next state *x*′ and goal *u* [23]. The intuition is that the passive dynamics (e.g. when navigating, the transition matrix given by a random walk through the environment) should act as a prior for the (goal-dependent) control dynamics, so that in the absence of an externally imposed goal, an agent should revert to the default policy.

**Figure 1:**
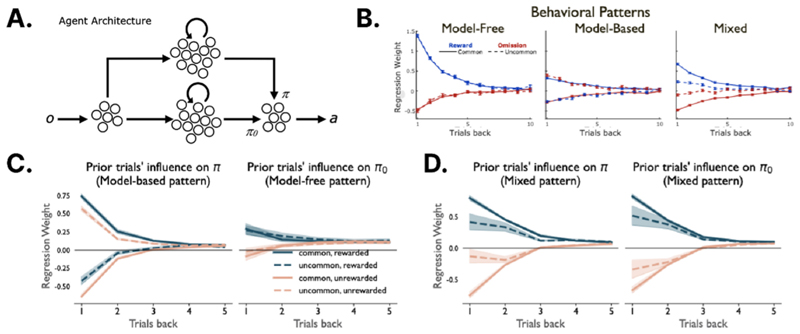
Dual-process models of cognitive flexibility. **(a)** Schematic diagram of the dual-process network architecture in Ref. [21••]. The lower pathway learns a default or habit-based policy via simplicity regularisation, whereas the upper pathway learns a policy that deviates from this to satisfy goals. **(b)** Behavioural data from the two-step task [22]. Logistic regression weights describing the influence on current-trial stage-1 choice (stay probability) of outcomes on the preceding five trials corresponding to (left) model-free, (middle) model-based and (right) mixed RL. **(c)** Same plots as in B but for (left) *π* and (right) *π*_0_. Patterns match respectively those previously described for model-based and model-free behaviour. **(d)** Same as Panel D but with different weighting of terms in the Minimum Descrption Length (MDL) objective (see Panel B, right). (d) Panels reproduced with permission from Ref. [21••].

Elaborating on this theme, one promising theory proposes a dual-process model of cognition grounded in deep RL [21••]. The idea is that a reward-maximising policy is jointly implemented by two distinct networks, which respectively learn a ‘habit-based’ default policy *π*_0_ and a ‘goal-based’ control policy *π* (Figure 1a). For the networks to adopt these distinctive roles via training from random weights, the system learns to maximise reward under two regularisation constraints: one that keeps *π*_0_ simple, and the other that tethers *π* to *π*_0_, so that the two policies do not diverge excessively. The first regulariser ensures that the habit-based network *π*_0_ is relatively *compressed* (encoded with fewer parameters or bits) and thus learns a policy that is simple and general, and that applies in a variety of circumstances. Theoretical work has shown that many cognitive phenomena, including stochasticity of choice, perseveration and chunking, can be rationally explained as a tendency to learn generalisable default tendencies via policy compression [24]. Relatedly, information-theoretic models of the PFC have argued for a subsidiarity principle, whereby organisms rely on the simplest policy possible for task execution, recruiting additional (and anatomically more anterior) control structures only when demanded by the context [25].

The second proposed regulariser penalises novel behaviours, ensuring that *π* does not stray too far from the default *π*_0_. It thus serves to keep cognitive flexibility from running amok, by preventing the network from dramatically overfitting to each goal in turn. Consistent with this idea, recent RL models have proposed that in addition to classical reward prediction errors (RPEs), the brain computes explicit penalties (called ‘action prediction error’ (APE) signals) when actions deviate from the norm encoded by a habit-based policy [26,27]. One recent recording study has even provided evidence for value-free teaching signals that resemble APEs in the tail of the mouse striatum, a region that does not receive dopaminergic RPE signalling [28]. More generally, the proposed division of labour between *π* and *π*_0_ resembles that observed in a biological agent equipped with twin habit- and goal-based systems. Indeed, the twin-network deep RL system trained was shown to capture a range of canonical behavioural phenomena that are touted as evidence for dual-process cognition in psychology and neuroscience, such as patterns of ‘two-step’ planning behaviour (Figure 1b–d), conflict-based interference and override of heuristic behaviours in classic judgement tasks [21].

Deep neural network models of task learning and control expose a ubiquitous problem for intelligent systems: that the brain needs constraints that trade off the merits of combining and separating task representations [29]. Neural networks trained via gradient descent from weights that are initially small in scale naturally learn to share task representations where possible (referred to as ‘low-dimensional’ neural coding), which confers robustness and supports behavioural generalisation [30,31]. However, representation sharing stands in tension with the need to learn distinct policies for divergent tasks. On the one hand, a pupil studying both Italian and Spanish can benefit from the shared orthography of words such as *casa* (house), *luna* (moon) and *triste* (sad), by learning representations that are shared between linguistic tasks.

However, excessive policy compression (here, the over-generalisation of Italian words to Spanish) would lead the student to make errors where vocabulary diverges, such as *cane* and *perro* (dog). In machine learning, training on multiple auxiliary tasks promotes the acquisition of shared representations, which improves transfer on a target task [32]. On the other, a bias towards representation sharing — encouraged, for example, by pressure to learn a control policy *π* that resembles the default *π*_0_, and thus to learn representations that are more generalisable — also offers a rational explanation for the costs of multitasking [33••], without having to invoke a nebulous notion of ‘mental resources’ [34].

## Deep meta-reinforcement learning

A well-known limitation of deep learning for explaining natural behaviour is that it offers only weak inductive biases, and thus necessitates training with implausible volumes of data over biologically unfeasible timescales [35]. One method that addresses this issue is called meta-learning [36]. In the context of RL, a neural network can be trained on a mixture of tasks invoking a broad distribution of triadic relationships among observations, actions and outcomes [37••,^38^]. This allows it to learn a strong inductive bias for solving new, unseen tasks — and indeed, there is a formal connection between this ‘meta-RL’ and Bayesian inference, with meta-training endowing the network with a ‘prior’ over tasks [39]. In combination with recurrent memory, meta-RL agents can learn a policy that adapts on the fly to entirely new tasks by ‘learning’ through adjustment of activation dynamics over trials. They thus learn ‘in context’ (based on the adaptation of recent activity patterns) and ‘in weights’ (through adjustment of tuneable parameters). Just as a neural network trained to respond ‘cat’ to many natural images of cats can generalise to previously unseen cats, a meta-RL system that learns a time-varying policy for solving 2-back and 4-back memory tasks can generalise this policy to solve a 3-back task on which it has not been trained, merely by inferring the correct task from the history of stimuli, actions and outcomes [40•].

**Figure 2:**
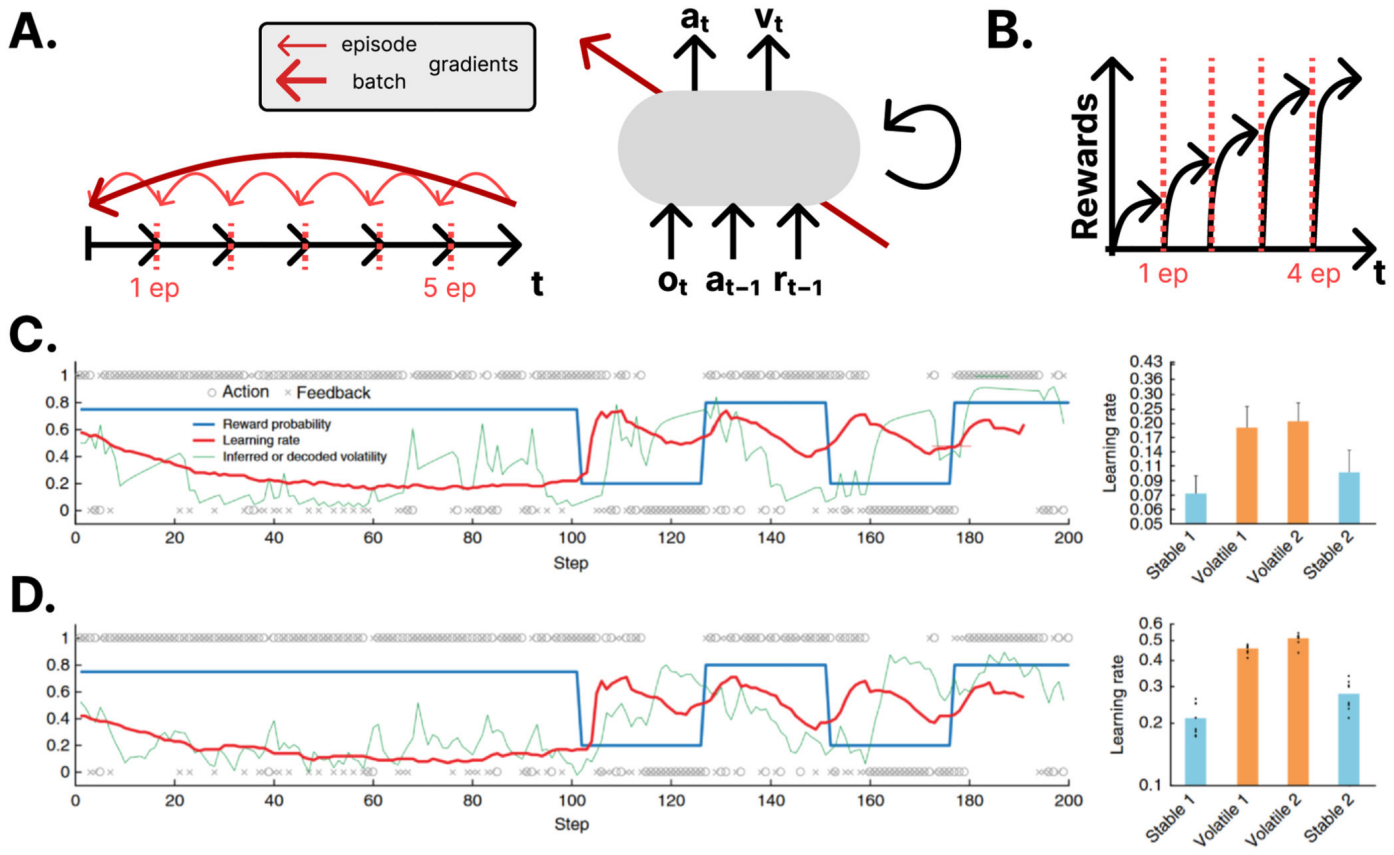
Deep meta-RL models of cognitive flexibility. **(a)** Overview over a meta-RL system. (left) The meta-RL is trained on a batch of individual episodes (black arrows separated by dotted red lines). The network is trained based on a meta-gradient (dark red) that is backpropagated through all of the episodes in a batch and takes into account the (light red) gradients on each of the individual episodes. (right) This meta-gradient (dark-red arrow) is used to train a recurrent neural network that uses an actor-critic architecture to output a distribution over policies and a value for a given state based on that time step’s observation as well as action, and reward received on the previous timepoint. **(b)** The resulting learning curve (black arrows) takes on a characteristic shape where performance is low at the beginning of every individual episode (separated by dotted red lines) as the agent learns to adapt more efficiently and flexibly to each subsequent episode. The behaviour closely matches those of the studied mice. **(c)** Example behaviour of a meta-RL agent in a two-alternative choice session reproduced from Hattori et al. [43]. **(d)** Mean optimality score that measures the optimality of action policy in this task considering the cumulative nature of reward availability, reproduced with permission from Ref. [43••].

The authors of one prominent paper (by Wang and colleagues, Figure 2a,b) explicitly linked meta-RL to recurrent neural dynamics in the primate prefrontal cortex [35], where longer time constants of neural integration may support flexible adaptation during sequential decision tasks [41]. Meta-RL may solve novel tasks by learning general-purpose maintenance and selection processes, perhaps implemented in PFC by gating mechanisms that rely on fast synaptic plasticity. Indeed, the underlying architecture on which this meta-RL system was based, the long short-term memory (LSTM) system, bears strong similarities to some computational theories of PFC function [41,42]. In fact, one recent paper has shown that blocking plasticity in rodent orbitofrontal cortex during meta-training on a bandit task disrupts the ability to deal with new contexts with different payout dynamics, consistent with the OFC supporting meta-training [43••] (Figure 2c,d). Using simulations, Wang et al. [37] showed that meta-RL networks are able to match the behaviour of biological systems on a variety of lab-based tasks that are typically thought to involve flexible control processes or planning, including assays of learning set [44] and multi-step inference [22]. This finding is striking because meta-RL is trained using model-free methods, and it shows how recurrent memory can be used to solve tasks that require flexible cognitive control.

## Deep learning models of meta-control

In biological agents, the ability to adapt flexibly to changing circumstances is linked to brain mechanisms that monitor for and respond to incipient conflict or errors. Neural signals in the medial PFC respond during the execution of inappropriate actions, even before any external reward or supervision is administered [45,46], and may be involved in more general regulation of thought and emotion [47]. These signals predict sub-sequent adjustment to behaviour, as if one dimension of cognitive flexibility is the engagement of control processes to cope with heightened mental demand [48,49] and with increased efficacy signifying with more impactful decisions [50•]. However, one major challenge for an agent in a stochastic environment is to learn policies that adapt optimally to the level of control that an agent has over the world. For example, if a tennis player’s repeated service faults are due to insufficient practice, then the player should spend more time on court; if they are due to high winds or an uneven surface, then more training will not help. Interestingly, there is evidence that even rodents may solve this complex credit assignment problem. For example, when learning a novel noisy visual stimulus discrimination task, rodents initially seem to devote undue time to the judgement rather than making rapid guesses to maximise reward rate, as if they were allocating resources to an attempt to improve their policy. Indeed, over the course of the whole task, the rats receive more reward than they would have under models that maximised reward rate initially [51,52••].

**Figure 3:**
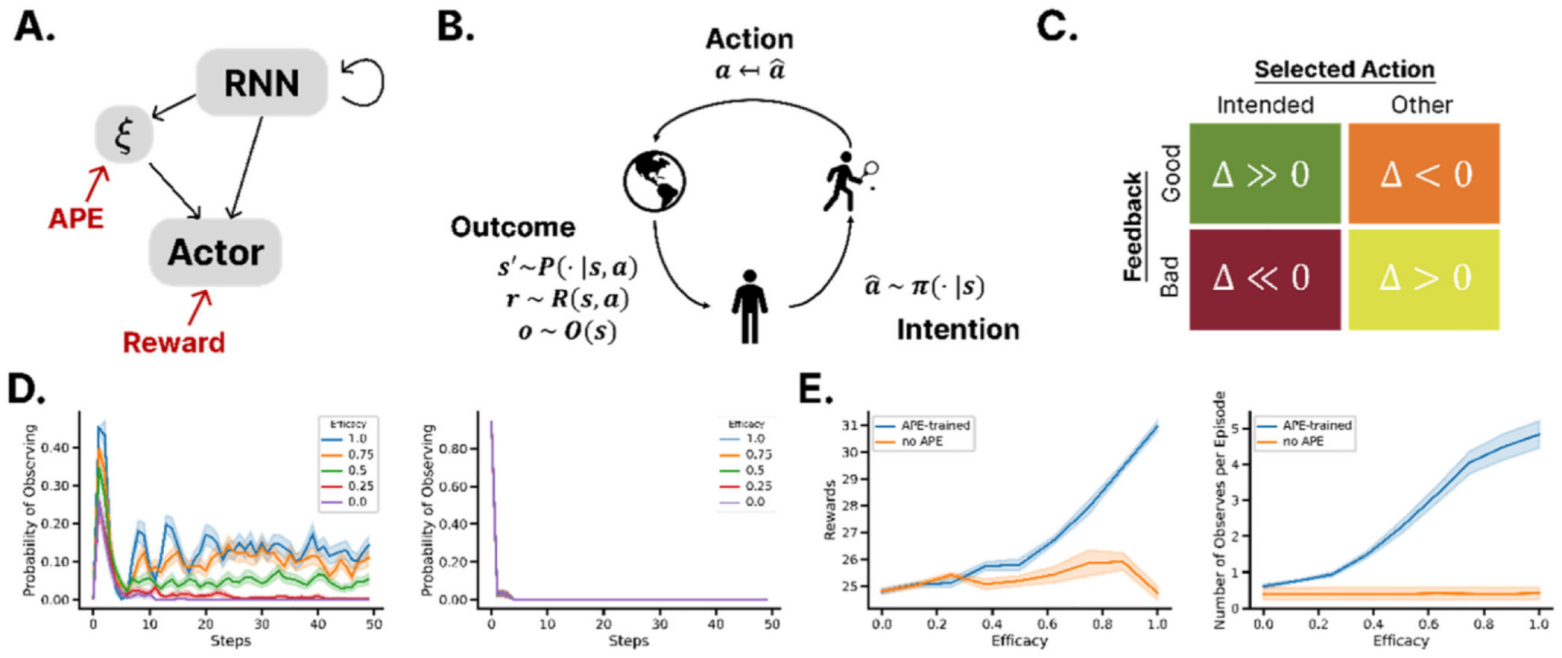
Deep learning models of meta-control. **(a)** Network architecture for the neural network model for self-control, in which a linear read-out of efficacy is trained from a recurrent learned state encoding and the two are jointly passed as inputs into an actor that decides on a policy. **(b)** The system is particularly useful in cases where action intention is not (necessarily) equivalent to the action that is ultimately taken and that determines the environmental outcome: this is the case both when there is internal motor noise and external variability such as wind affecting a tennis ball. These situations can be modelled as a two-step task. **(c)** Similar to a two-step task, the potential for discrepancies between chosen and intended outcome results in counter-intuitive update structures in which negative feedback from the environment does not necessarily mean that the actor should downweigh an intended action, but rather should work to improve its execution. **(d)** Policy over an episode for (*left*) a sample APE-trained model and (*right*) a non-APE-trained model. **(e)** Behaviour per efficacy level across five model instantiations in terms of (*left*) reward per episode and (*right*) number of trials on which the users choose to observe.

In a similar vein, one recent study showed that vanilla deep policy gradient networks, implemented as LSTMs, struggle to meta-learn policies that adapt according to the level controllability in the environment. Networks were meta-trained on multiple versions of an ‘observe or bet’ paradigm [53–55] (Figure 3 a), a paradigm that forces the agent to decide on each trial between observing the outcome of a bandit without being rewarded (observe) or choosing among bandits without viewing any reward obtained (bet). On each block, there was a fixed probability that the action chosen on ‘bet’ trials was randomly perturbed (such as a tennis player at the mercy of the elements; Figure 3 b); intuitively, if this probability is 100%, then observation is pointless, because your choices never translate into their intended consequences (you have zero ‘efficacy’). Whilst standard meta-trained agents failed to learn to adapt their policy according to efficacy levels or perturbation (Figure 3 c), networks that were additionally trained to predict their own efficacy via a single additional unit, thereby learning a ‘sense of agency’ [56], succeeded and mirrored human performance on the task [57••] (Figure 3 d-e). The same results hold true in a second task in which participants have access to an additional action (‘sleep’) that allows them to increase their efficacy, but with higher levels of environmental efficacy now corresponding to lower levels of control-seeking behaviour.

Analysis of neural coding in the networks able to exert meta-control revealed that they explicitly represented levels of efficacy along the first two principal components of neural activity of the LSTM layer, whereas the purely reward-driven network did not. This differentiation may allow the networks to assign credit differentially depending on whether an error occurs, as humans can [58•]. This motivates the existence of dedicated neural systems, such as those in the medial PFC, that engage in error monitoring, and serve to detect mismatches between intended and executed actions [59].

## Conclusions: cognitive flexibility in deep networks

More recently, the remarkable power of in-context learning has been demonstrated in transformer-based models trained on autoregressive problems, including learning to execute simple programmes with novel inputs [60–62], and generating plausible responses to natural language queries [63]. Large language models (LLMs) are typically fine-tuned with a method called reinforcement learning from human feedback (RLHF), which is a form of inverse RL that is used to nudge the model towards more helpful and less-harmful replies [64]. Most implementations of RLHF use proximal policy optimisation, an RL method in which the updated policy is tethered to the existing one, so that fine-tuned language models do not stray too far from the pretraining distribution [65]. This is reminiscent of how *π* is regularised towards *π*_0_ in the dual-process networks above.

The deep neural network models described in this review are trained with trial and error. Nevertheless, they are able to recreate many behaviours that cognitive scientists have thought to require explicit mechanisms for explicit planning and mental simulation, and have ascribed to dedicated subsystems for goal-based cognition or model-based inference. LLMs provide the most vivid example of this phenomenon. RLHF is an offline training method that is applied before networks are frozen and deployed. Remarkably, however, its benefits seem to transfer widely across settings — for example, fine-tuning in English seems to generalise readily to other, lower-resource languages [66]. After fine-tuning, leading LLMs seem to be able to reliably solve (previously unseen) reasoning, maths and coding problems that are classical hallmarks of ‘system-2’ cognition. This shows that rather than requiring explicit rollouts through a model of the world, these tasks can be solved by ‘habit-based’ fine-tuning of neural networks that have acquired rich semantic knowledge through diverse and voluminous pre-training. These feats of in-context learning (or meta-learning) thus invite us to revisit traditional claims about the computational basis for habit-based (stable) and goal-based (flexible) cognition. It turns out that expressive neural networks, trained by trial and error alone, can show much greater levels of cognitive flexibility than was previously thought possible.

The application of deep neural networks to modelling cognitive flexibility is in its infancy. There are several promising avenues for future research, including dual-process neural networks, deep meta-RL and networks capable of learning meta-control. To date, these approaches have been pursued independently, but they are potentially complementary, and could be combined. An interesting avenue for further research is to focus on how RL can be combined with generative modelling to produce high-performing models that learn from both observations and rewards [67,68]. Insights from the training of large generative models with RL may help us understand how richness of prior experience promotes cognitive flexibility in humans.
